# Cysteinyl Leukotrienes as Potential Pharmacological Targets for Cerebral Diseases

**DOI:** 10.1155/2017/3454212

**Published:** 2017-05-10

**Authors:** Paolo Gelosa, Francesca Colazzo, Elena Tremoli, Luigi Sironi, Laura Castiglioni

**Affiliations:** ^1^Centro Cardiologico Monzino IRCCS, Via Carlo Parea 4, 20138 Milan, Italy; ^2^Department of Pharmacological and Biomolecular Sciences, University of Milan, Via Giuseppe Balzaretti 9, 20133 Milan, Italy

## Abstract

Cysteinyl leukotrienes (CysLTs) are potent lipid mediators widely known for their actions in asthma and in allergic rhinitis. Accumulating data highlights their involvement in a broader range of inflammation-associated diseases such as cancer, atopic dermatitis, rheumatoid arthritis, and cardiovascular diseases. The reported elevated levels of CysLTs in acute and chronic brain lesions, the association between the genetic polymorphisms in the LTs biosynthesis pathways and the risk of cerebral pathological events, and the evidence from animal models link also CysLTs and brain diseases. This review will give an overview of how far research has gone into the evaluation of the role of CysLTs in the most prevalent neurodegenerative disorders (ischemia, Alzheimer's and Parkinson's diseases, multiple sclerosis/experimental autoimmune encephalomyelitis, and epilepsy) in order to understand the underlying mechanism by which they might be central in the disease progression.

## 1. Introduction

Growing evidence indicates that cysteinyl leukotrienes (CysLTs), a group of highly active lipid mediators, synthetized from arachidonic acid via the 5-lipoxygenase (5-LOX) pathway, play a pivotal role in both physiological and pathological conditions.

Cysteinyl leukotrienes—LTC4, LTD4, and LTE4—exhibit several biological activities in nanomolar concentrations through at least two specific G protein-coupled receptor (GPCR) subtypes named CysLTR-1 and CysLTR-2 which show 38% homology [1]. These endogenous mediators show different affinity toward their receptors [2]: LTD4 indeed is the most potent ligand for CysLTR-1 followed by LTC4 and LTE4 [3], whereas LTC4 and LTD4 equally bound CysLTR-2, while LTE4 shows only low affinity to this receptor [1]. However, the biological effects of CysLTs do not seem to be mediated only by CysLTR-1 and CysLTR-2. Indeed, these receptors are phylogenetically related to purinergic P2Y class of GPCRs [4] and evidence reported in the literature suggests the existence of additional receptors responding to CysLTs [5], such as GPR17 [6], GPR99 [7], PPAR*γ* [8], P2Y6 [9], and P2Y12 [10].

**Figure 1:**
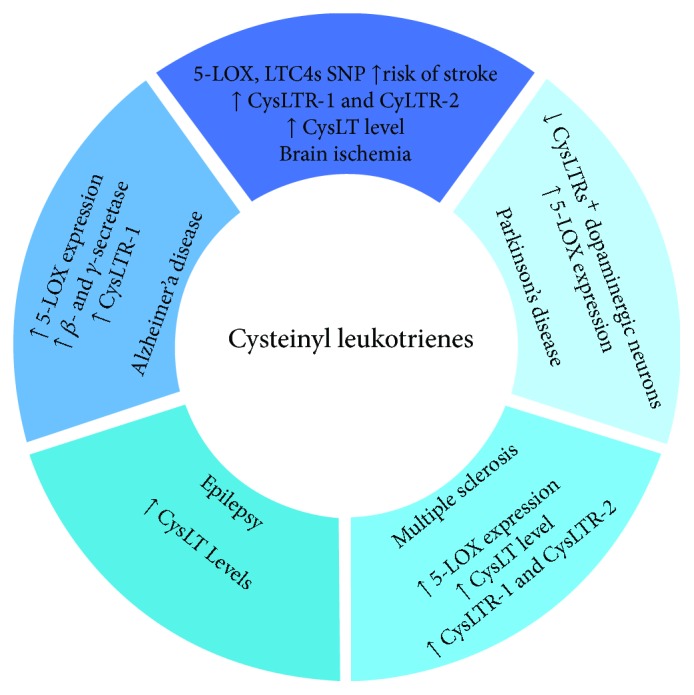
CysLTs in neurodegenerative diseases. The circle shows the changes of the CysLT pathway components grouped for the different neurodegenerative diseases and observed in human patients and in in vitro/in vivo models.

In the last decade, several lines of evidence link CysLTs, central in the pathophysiology of respiratory diseases, such as asthma and allergic diseases [11–14], to other inflammatory conditions including cancer and cardiovascular, gastrointestinal, skin, and immune disorders [15, 16]. Among them, a role of CysLTs and their receptors has been emerging in central nervous system (CNS) diseases, such as cerebral ischemia [15, 17, 18], intracerebral hemorrhage [19], brain trauma [20, 21], epilepsy [22], multiple sclerosis [23], Alzheimer's disease [24], and brain tumor [25]. This review will summarize the state of present research about the involvement of CysLT pathway (Figure 1) and the effects of its pharmacological modulation (Table 1) on CNS disorders.

## 2. Cerebral Localization of CysLT Receptors

In healthy brain, the expression of the CysLTRs is weak, but it was reported to increase during several pathological conditions [15, 17, 20]. CysLTR-1 [26], whose expression is normally lower than the CysLTR-2 one [1, 3], is localized in microvascular endothelial cells [21], in glial cells, and in several types of neuronal cells [15, 27, 28].

In human brain, the CysLTR-2 is expressed in many regions, such as hypothalamus, thalamus, putamen, pituitary, and medulla [1] by vascular smooth muscle cells [20] and by astrocytes [18]. After brain trauma and in brain tumors, it was also observed in neurons and in glial-appearing cells [20].

Glial cells, namely astrocytes and microglia, are key players in inflammation typically associated with neurodegenerative diseases, and their functions are regulated in a CysLTR subtype dependent manner [18, 28, 29]. Through CysLTRs localized on glial cells, CysLTs may mediate not only crucial reparative responses in the acute phase [30] but also detrimental effects in the chronic phase [31] of brain damage. Moderately activated microglial cells play a neuroprotective role due to their ability to remove dead cells, to release trophic factors, and to contribute to angiogenesis, neurogenesis, and axonal remodelling [32, 33], promoting reorganization of neuronal circuits and improving neurological recovery [34]. However, when overactivated, microglia show important adverse effects by releasing detrimental factors [35, 36] such as cytokines and nitric oxide (NO) [37] and by activating inflammation-related kinases and transcription factors [38]. Similarly, astrocytes are known to exert a protective function during brain injury [39, 40], but astrogliosis may contribute to neuronal injury [41–44].

Data indicate that in microglia, both CysLTs and CysLTRs participate in the inflammatory response [45, 46]; nevertheless, the impact of CysLTR-1 and CysLTR-2 in the process is controversial. A number of in vitro evidence indicate a relevant role of CysLTR-1 in microglial activation. It was reported that rotenone—used in generating animal models of Parkinson's disease (PD)—increased CysLTR-1 expression in mouse microglial BV2 cell line [47, 48] and that treatment with the CysLTR-1 antagonist montelukast prevented phagocytosis and cytokine release [48]. Moreover, the activation of mouse microglial BV2 cells seems to be greatly mediated by CysLTR-1 than CysLTR-2 [28]. On the other hand, another study showed that, in primarily cultured microglia, the CysLTR-2 resulted the main regulator of microglia activation. Indeed, the CysLTR-2 antagonist HAMI 3379 inhibited phagocytosis and cytokine release induced by oxygen-glucose deprivation/reperfusion (OGD/R) and by LTD4, whereas montelukast was effective only against OGD/R [46].

These conflicting results suggest that the responses mediated by CysLTR-1 and CysLTR-2 may change across experimental conditions; nevertheless, the role of CysLTR-2 in the regulation of microglial activation and phagocytosis is supported by in vivo evidences. Indeed, the CysLTR-1 antagonist pranlukast did not reduce the accumulation of microglia in the ischemic cerebral cortex [49], while HAMI 3379 significantly attenuated the number of microglia in the ischemic core and in the boundary zone [50].

Unlike in microglia, the function of each CysLTR subtype in *astrocytes* is already clear. A number of evidence support the major role of CysLTR-1 in regulating astrocyte activation, suggesting its involvement in astrocytosis and in glial scar formation. In vitro, astrocyte proliferation, induced by low concentrations of LTD4 or by mild OGD, is indeed mediated by CysLTR-1, but not by CysLTR-2 [29]. The CysLTR-1 also participates in astrocyte migration induced by transforming growth factor-*β*1 (TGF-*β*1) and LTD4 [51]. In fact, this event was attenuated by administration of the CysLTR-1 antagonist montelukast, but not by the CysLTR-2 specific antagonist Bay CysLT2 [51].

### 2.1. Brain Ischemia

A strong indication for the involvement of the leukotriene-synthesizing pathway in the occurrence and evolution of ischemic brain diseases comes from genetic studies. In humans, a genetic variant of the gene ALOX5AP, encoding 5-lipoxygenase activating protein (FLAP), is associated with two times greater risk of stroke by increasing leukotriene production and inflammation [52–56]. The −444 A/C polymorphism on the LTC4 synthase gene also predicts an increased risk for ischemic cerebrovascular disease [57, 58]; conversely, the −1072 G/A polymorphism of the same gene results in decreased risk of ischemic cerebrovascular disease [57]. Nevertheless, to date, the meaning of these polymorphisms in the brain ischemia has not been fully understood; thus, a comprehensive analysis of these gene polymorphisms is required.

**Figure 2:**
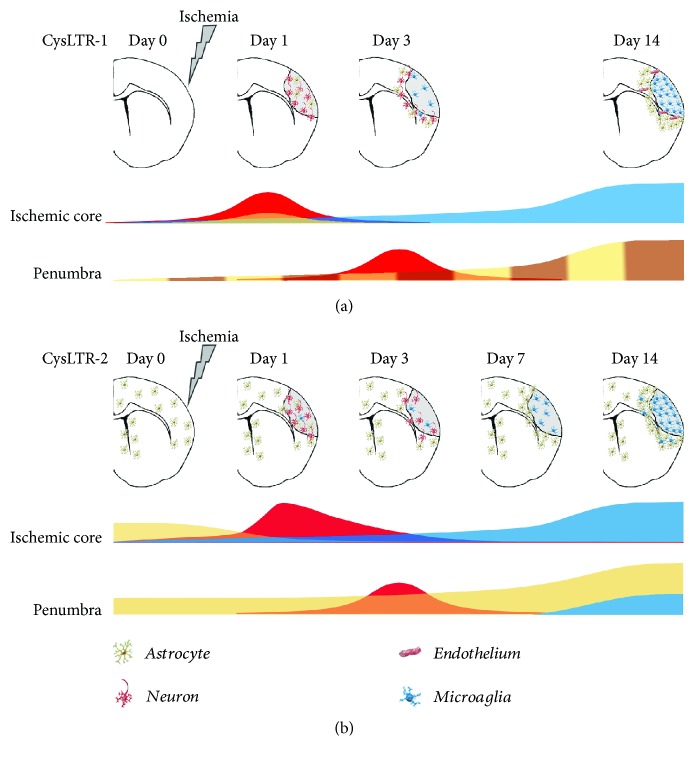
Spatio-temporal expression of the CysLT1 and CysLT2 receptors after focal cerebral ischemia in rodents. (a) In the control brain, CysLT1 receptor is weakly expressed (time 0) [15, 61]. Following middle cerebral artery occlusion (MCAo), its expression, at the ischemic core level, is biphasic: at day 1 postischemia, the receptor is mainly expressed in neurons (red wave) [15, 60, 61] and, to a lesser extent, in astrocytes (orange) [15]; between 7 and 14 days postischemia, it increases in microglia (blue) [15]. In the boundary zone, that is, the “*penumbra*,” the receptor's expression is mainly expressed in neurons (red wave) at 3 days [60] and then it increases over time in most hypertrophic astrocytes (yellow) [15] and microvascular endothelial cells (brown) [15], reaching a peak after 14 days. (b) In the healthy brain, the CysLT2 receptor is primarily expressed in GFAP^+^ astrocytes around the lateral ventricles and in the cortex [18]. In the ischemic core, one day postischemia, the expression of CysLT2 receptor shows a rapid and transient peak in neurons (red) [18, 60] and then gradually disappeared over 3 days. In the hypertrophic microglia (blue), it slowly increases over time and reaches a peak after 14 days [18]. In the *penumbra* (boundary zone), following its induction at day 0, the receptor's expression is mainly expressed in neurons (red wave) at 3 days [60] and then it increases over time in astrocytes [18]. After one week, its expression also increases in the microglia [18].

Data from in vivo and in vitro studies show that the production of CysLTs increased in the brain of rodents that underwent a cerebral ischemic insult [38] and in primary culture of neurons [59] and astrocytes [29] subjected to OGD. In rat that underwent middle cerebral artery occlusion (MCAO), the brain levels of CysLTs reached the peak within 3 hours and remained high for at least 24 hours [38]. Consequently, also the expression of CysLTR-1 and CysLTR-2 was upregulated in injured neurons during the acute phase (about 24 hours) and in activated microglia and proliferating astrocytes [15, 17, 18, 60, 61] during the late phases (3–28 days) (see Figure 2). Taken together, these findings suggest that CysLTs could mediate the acute ischemic neuronal injury and the subsequent secondary injury mainly by promoting microgliosis and astrocytosis.

Although the role of CysLTs in brain ischemia is supported by several evidences, the mechanisms through they mediate neuronal injury are not fully clarified. Indeed, in vitro culture of neuron-like PC12 cells transfected with CysLTR-1 and CysLTR-2 showed distinct sensitivities to ischemic injury, which resulted prominent in CysLTR-2-transfected cells [62], but neither CysLTR-1 nor CysLTR-2 were able to directly induce neuronal injury [46, 63]. Moreover, OGD/R-induced ischemic injury was not attenuated by the selective CysLTR-2 antagonist HAMI 3379 and by CysLTRs RNA interference in primary neurons [46]. Conflicting results were obtained by using the CysLTR-1 antagonist montelukast: this drug had no effect on neuronal viability [63] and an only moderate effect on the neuronal morphologic changes after OGD [64], while in another study improved viability in OGD/R neurons [46].

Overall, these data suggest that the direct effect of CysLTs on neurons causes only a mild type of injury; nevertheless, CysLTs could indirectly mediate a more severe neuronal injury in the presence of complex intercellular interactions. Indeed, in neuron-microglial cocultures, LTD4 was shown to induce neuronal injury [46]. Conditioned medium from microglia pretreated with OGD/R and LTD4 also induced neuronal injury that was inhibited by HAMI 3379 and CysLTR-2 short hairpin RNA (shRNA), more potently than montelukast. These findings demonstrated the main role of microglial CysLTR-2 in the induction of neuronal death compared to CysLTR-1 [46].

On the contrary, the role of CysLTR-1 and CysLTR-2 in astrocyte-mediated neuronal injury is still unclear. In vitro, CysLTR-1 mediates astrocyte proliferation after mild ischemia, whereas CysLTR-2 mediates astrocyte death after more severe ischemia [29]. However, in neuron-astrocyte cocultures, subjected to OGD/R and LTD4 exposure, CysLTR-1 and CysLTR-2 antagonists were unable to completely prevent astrocyte-mediated neuronal necrosis [46]. Astrocyte reactivity seems instead to be mainly mediated by CysLTR-1 rather than CysLTR-2. Indeed, CysLTR-1 was involved in glial scar formation during the chronic phase after focal cerebral ischemia [15, 65], and CysLTR-1 antagonist, but not CysLTR-2, was able to reduce the astrocyte response in the subacute phase after brain ischemia [50].

Together with microglia and astrocytes, also endothelial cells seem to contribute in CysLTR-mediated brain injury. The CysLTR-1 is highly expressed in microvascular endothelia at the ischemic boundary zone in rat [15] and in brain tissue after trauma in human [21]. Furthermore, CysLTs induced the disruption of blood-brain barrier (BBB) and the subsequent development of cerebral edema, whose progression was attenuated by CysLTR-1 and CysLTR-2 antagonists [66–69]. These data suggest that CysLTR antagonists may be critical in modulating the function of cerebral microvascular endothelia and in preserving the integrity of BBB against cerebral insults.

Overall, these findings lend support to the hypothesis that a pharmacological modulation of CysLT pathway can open new terrain for therapeutic approaches targeted at attenuating local inflammation in order to modulate its impact in cerebral ischemia.

#### 2.1.1. Inhibitors of FLAP/5-LOX

The first in vivo experimental evidence of neuroprotection through postischemic modulation of LT levels was obtained by using AA-861, a selective inhibitor of 5-LOX, in a model of transient ischemia in gerbils [70, 71]. This effect was confirmed in a model of permanent MCAO by the use of MK-886 and zileuton, selective inhibitors of FLAP and 5-LOX, respectively. MK-886 decreased the acute infarct size [72], whereas zileuton attenuated neurological dysfunction and cerebral infarction, probably inhibiting inflammatory reaction, neuronal apoptosis, and BBB disruption [73–75]. Nevertheless, despite these promising results, the association between LTs and brain ischemia is not fully demonstrated. In fact, conflicting results were obtained by using models of FLAP or 5-LOX knockout mice since one study reported an improvement of stroke damage in FLAP knockout mice [76] whereas another one showed no difference in the infarct size between 5-LOX knockout and wild-type MCAO mice [77].

#### 2.1.2. CysLTR-1 Antagonists

Despite the evidence that CysLTR-2 is the main CysLTR subtype in the normal brain, the lack of selective CysLTR-2 antagonists limited, for long time, the clear understanding of the role of CysLTR-2 in cerebral injury. Hence, the first line of data, from experiments carried out with CysLTR antagonists, were limited to CysLTR-1. Pranlukast inhibited acute, subacute, and chronic ischemic injury in the brains of mice and rats after focal cerebral ischemia [15, 49, 65, 78]. Moreover, the postischemic treatment with pranlukast exerted a long-term protective effect in MCAO mice, attenuating the lesion volume, increasing the neuron density, inhibiting the ischemia-induced glial scar formation, and finally improving the neurological deficits and the motor-sensory recovery [65]. Montelukast attenuated infarct volume, brain atrophy, neuron loss, and behavioural dysfunction after focal cerebral ischemia in both mice and rats [6, 79, 80]. It also exerted prophylactic effects in global cerebral ischemia/reperfusion injury, decreasing infarct size, oxidative stress, inflammation, release of glutamate, apoptosis, and lactate dehydrogenase activity [81]. In neonatal hypoxic-ischemic rats, montelukast showed neuroprotective effects, likely inhibiting apoptosis through the increase of TERT, the catalytic center of the telomerase complex, and Bcl-2 [82].

In summary, two possible mechanisms could be responsible in mediating the effect of CysLTR-1 antagonists on cerebral ischemia: (i) the reduction of BBB disruption and inflammation in the acute/subacute phases [15, 68, 83] and (ii) the attenuation of astrocyte proliferation and related glial scar formation in the chronic phase [29, 65].

#### 2.1.3. CysLTR-2 Antagonists

Recently, Bay CysLT2 and HAMI 3379 have been reported to selectively antagonize CysLTR-2 [84, 85]. The intracerebral ventricular (i.c.v.) injection of HAMI 3379 showed to protect against acute brain injury in MCAO rats. This treatment attenuated neurological deficits and reduced lesion volume, edema, and neuronal degeneration [69]. HAMI 3379 was also effective when intraperitoneally administered within 1 hour after ischemia in MCAO rats [50]. In the acute phase, HAMI 3379 attenuated neuronal loss, improved neurological score, and reduced cytokine levels in serum and cerebrospinal fluid, and in the late phase, it strongly decreased the microglia/macrophage-associated postischemic inflammation, without affecting astrogliosis. The effect of the CysLTR-2 antagonists on acute ischemic brain injury could be explained by at least four possible mechanisms: (i) a direct protective action on neurons [62]; (ii) protection to astrocytes, since it was reported that in severe ischemic injury, the activated CysLTR-2 could induce astrocyte death [29]; (iii) prevention of the development of cytotoxic edema [69], effect that in astrocytes is mediated by upregulating the water channel protein AQP4, which is induced by LTD4 [86] and by ischemia-like injury [87]; and (iv) attenuation of microglial activation [50]. Potential interactions between CysLTR-1 and CysLTR-2 need also to be considered. Indeed, it was reported that CysLTR-2 could limit the formation of CysLTR-1 homodimers and control its cellular surface expression [88, 89].

#### 2.1.4. The CysLTR-Independent Effects

Despite the evidence of a direct involvement of CysLTRs in brain ischemia, we cannot rule out that the neuroprotective effects could be partially ascribed to CysLTR-independent mechanisms. Indeed, it is reported how part of the effects of CysLTs are mediated by GPR17. This receptor is phylogenetically related to CysLTRs [6, 90, 91], activated by endogenous cysteinyl leukotrienes (LTD4 and LTC4) [6, 92] and inhibited by the CysLTR-1 antagonist montelukast [6, 90]. The GPR17 colocalizes and dimerizes with CysLTR-1 and negatively regulates CysLTR-1-mediated effects [93, 94]. It was also upregulated in damaged tissues [6], and the knockout of GPR17 reduced neuronal injury after ischemia [90, 95]. Moreover, in differentiated PC12 cells, the knockdown of GPR17 abolished LTD4-induced effect on cell viability [96].

Restricting to montelukast, its neuroprotective CysLTR-1-independent effects could be also due to its ability to inhibit phosphodiesterases (PDEs) [97]. Indeed, the decreased activity of PDEs may be beneficial to ischemic neuronal injury, since the resultant accumulation of cAMP protects neurons from ischemic brain injury [98, 99] and inhibitors of PDEs have protective effects on neurons [100, 101]. In addition, montelukast was shown to inhibit P2Y receptors [9, 102, 103] and oxidative stress [104–106], which is the major cause of the ischemic injury [107–109]. Taken together, these data add new evidences for the neuroprotective effects of CysLTR-1 antagonists and highlight the need for further studies that will define the possibility to use CysLTR-1 antagonists for treatment of stroke patients. Up to now, there is only a recent cohort study that showed a reduced risk for stroke associated with montelukast use in patients with a prior stroke [110].

### 2.2. Alzheimer's Disease

Alzheimer's disease (AD) is the most common aging-associated neurodegenerative condition resulting in progressive loss of memory and cognition and affecting worldwide over 35 million of individuals [111]. It is pathologically characterized by extracellular deposit of *β*-amyloid (A*β*) plaques and intracellular neurofibrillary tangles (NFTs) of tau protein [112, 113]. Altered inflammatory reactions and dysregulation of inflammatory cytokines as well as immune cell (i.e., microglia and astrocytes) activation are also strongly associated with AD pathology and cognitive dysfunction [114, 115].

Postmortem studies have shown that 5-LOX expression is upregulated in human brain of AD patients [116, 117]. Experiments on animal models have provided evidence on the relevant role of 5-LOX in the development of AD. In detail, the overexpression of this enzyme resulted in a worsening of amyloidosis in Tg2576 mice [118] and in an exacerbation of memory deficits, amyloid plaques, and tau tangles in triple transgenic mice (3xTg-AD) [119]. Of note, these 5-LOX-induced effects seem to be mediated by an increase of *γ*-secretase complex [119]. The direct involvement of 5-LOX in the *γ*-secretase pathway is confirmed by findings of both genetic and pharmacological inhibition of 5-LOX that reduced the activity of *γ*-secretase [117, 120, 121]. The increase of *γ*- and *β*-secretase occurs also in the presence of leukotriene metabolites of 5-LOX, such as 5-HPETE, LTC4, and LTD4 [117, 122]. Furthermore, in vivo and in vitro studies showed that LTD4-induced upregulation of CysLTR-1 is correlated with increased A*β* and amyloid precursor protein (APP) and with cognitive dysfunctions in mice [122–124]. In parallel, the microinfusion of A*β*_1–42_, a more neurotoxic A*β* species, resulted in significant increase in CysLT1-R expression in the hippocampus and cortex [125].

Genetic ablation of 5-LOX clearly reduced A*β* brain deposition in Tg2576 mice and in dexamethasone-induced A*β* mice [117, 126], while pharmacological studies using specific FLAP and 5-LOX inhibitors, MK-591 and zileuton, supported the genetic knockout findings showing in vivo ameliorative effect on AD phenotypes [120, 121, 127, 128].

The inhibition of 5-LOX also exerts beneficial effects on AD pathology-induced oxidative and inflammatory insult. In cultured rat hippocampal neurons, the pharmacological 5-LOX pathway inhibition resulted in reduced A*β*-induced reactive oxygen species generation [129]. Tg2576 mice receiving MK-591 showed a reduction in brain levels of IL-1*β* and in the immunoreactivity for CD45, a marker of microgliosis, and GFAP, a marker of astrogliosis [120].

Data indicate that pathological AD symptoms are attenuated through administration of selective CysLTR-1 antagonists such as pranlukast and montelukast. In primary culture of mouse neurons, A*β*_1–42_ markedly increased CysLTR-1 expression, which was associated with cytotoxicity, inflammatory, and apoptotic responses. Incubation with pranlukast and montelukast reversed the upregulation of A*β*_1–42_-induced CysLTR-1 and NF-kB p65 and activated caspase-3 expression and the downregulation of Bcl-2 [130,131]. In bilateral i.c.v. A*β*_1–42_-injected mice, pranlukast and montelukast reversed the A*β*_1–42_-induced cognitive deficits associated to inflammatory and apoptotic responses, as evidenced by decreased NF-kB p65, TNF-*α*, IL-1*β*, and caspase-3 in the hippocampus and cortex [125, 132]. Moreover, in other studies, montelukast restores learning and memory function in old rats, in which cognition is compromised and the hippocampus concentrations of 5-LOX transcripts and of leukotrienes were increased [27, 133]. Although the inhibition of CysLTR-1 could explain the maintained BBB integrity and the reduced age-associated neuroinflammation, in particular microglial reactivity, the authors suggest that montelukast promotes hippocampal neurogenesis, in particular progenitor cell proliferation, most likely through blocking GPR17 [27].

### 2.3. Parkinson's Disease

Parkinson's disease (PD) is a common neurodegenerative disease, characterized by the depletion of striatal dopamine due to degeneration of dopaminergic neurons in the substantia nigra of the brain and manifested by the movement disorders in elderly populations. Brain inflammation and oxidative stress were reported to play important roles in the pathogenesis of PD [134–136].

Recent evidences suggest an involvement of 5-LOX in nigrostriatal dopaminergic injury. Indeed, 5-LOX upregulation was shown in MPTP-induced animal model of PD [137] and the overactivation of the 5-LOX pathway may lead to neurodegeneration by lipid peroxidation [138]. On the contrary, the inhibition of 5-LOX attenuates LPS-induced oxidative stress and dopaminergic neurodegeneration [139]. Furthermore, MK-886 treatment antagonized the MPP^+^-induced toxicity of dopaminergic neurons in SH-SY5Y cell line, a common cellular model for PD, and in midbrain neuron-glia cocultures [137]. Of note, LTB4, but not LTD4 or 5-HETE, enhanced the MPP^+^-induced cytotoxicity in the rat midbrain culture. MK-866 protects also neurons against MPTP-induced neurotoxicity in mice [137].

A recent study reported that CysLTR-1, CysLTR-2, and GPR17 are localized in dopaminergic neurons of healthy mouse brain [140]. In MPTP-treated mice, the number of CysLTR-1^+^, CysLTR-2^+^, and GPR17^+^ dopaminergic neurons was significantly reduced, suggesting an involvement of these receptors in this animal model of PD.

### 2.4. Multiple Sclerosis/Experimental Autoimmune Encephalomyelitis

Multiple sclerosis (MS) is a chronic inflammatory neurological disease of the CNS, characterized by recurrent and progressive autoimmunity-mediated demyelination, and resulting in severe infiltration of CD4^+^ T cells, development of sclerosis, oligodendrocyte damage, and, ultimately, axonal loss [141, 142]. Brain atrophy, one of the major features of the disease, occurs in the advanced stage of the disease [143].

The role of arachidonic acid cascade in the demyelination of the CNS was suggested by studies utilizing animal models of experimental autoimmune encephalomyelitis (EAE) [144, 145]. Microarray analysis studies indicated that the mRNA of 5-LOX is upregulated in brain lesions of patients with primary progressive and with relapsing-remitting MS (RRMS) [146] and in the peripheral blood cells of patients with RRMS during the relapse and the remission phases [147]. These results are corroborated by data obtained with immunohistochemistry analysis showing the presence, in the active and chronic inactive inflammatory lesions, of macrophages strongly positive for 5-LOX staining [146]. Gene and protein expressions of 5-LOX are also increased in CNS of experimental autoimmune encephalomyelitis (EAE) [146, 148] and cuprizone-treated mice [149], the widely used animal models utilized to mimic demyelination and MS.

Notably, the concentration of 5-LOX-derived LTB4, but not of CysLTs (LTC4, LTD4, and LTE4), was significantly increased in CSF of patients with clinically active MS [150]. Contrary, previous studies reported higher levels of LTC4 in the CSF of MS patients likely due to the less accurate analytical techniques utilized [150, 151]. In EAE mice, the CysLT levels in both serum and CSF were significantly increased after disease onset, whereas did not change significantly in the brain and spinal cord, although the trends of increase could be observed [148]. Moreover, LTD4 showed a dose-dependent chemotactic activity on splenocytes, in particular those of CD4^+^ cells, from EAE mice [148].

The CysLTR-1 and CysLTR-2 expression was found to be upregulated in the brain after disease onset in EAE mice [148]. CysLTR-1 started to increase from the onset of the disease and kept increasing throughout the whole process also in spinal cord.

There are several evidences that 5-LOX pathway blockade could ameliorate the pathological development of MS. In EAE mice, the blockade of the cytosolic phospholipase A2*α* and of its downstream enzyme 5-LOX was found to ameliorate the disease pathogenesis during the effector phase of EAE [152] and to delay the onset and reduce cumulative severity of the pathology [153]. Although MK-886 did not attenuate demyelination in cuprizone-treated mice, the pharmacological inhibition of 5-LOX improved axonal damage and motor deficits related to MS pathology [149].

CysLTR-1 antagonists montelukast and zafirlukast were shown to ameliorate clinical symptoms in EAE mice [148]. In detail, montelukast reduced the demyelination and leukocyte infiltration in the spinal cord sections, the secretion of IL-17 from myelin oligodendrocyte glycoprotein-specific T cells, the permeability of the BBB, and the chemotaxis of T cells. Interestingly, montelukast was still able to reduce the severity of EAE when given after the onset of the disease, suggesting, in addition to the preventive effect, also a possible therapeutic benefit of this drug. Relevantly, the infiltration of Th1^+^ and Th17^+^ cells in the inflamed area of the brain was reduced by the dual inhibitor of LOX/COX pathway flavocoxid and by montelukast in EAE mice [148, 154].

Finally, since GPR17 was found to be reexpressed or upregulated in demyelinating lesions in EAE and human MS plaques [155], GPR17 and purinergic signalling has been strongly suggested as targets for new reparative approaches in MS [155–157].

### 2.5. Epilepsy

Accumulating clinical and experimental evidence suggests that inflammatory mediators play a relevant role in the pathophysiology of epilepsy [158, 159]. Nevertheless, only few studies have investigated the role for LOX-derived arachidonic acid metabolites in epilepsy [160–162]. Leukotriene levels were found to increase in a time-dependent manner in the brain during kainate-induced seizures in rats [160], and LTD4 i.c.v. injection facilitated pentylenetetrazol- (PTZ-) induced seizures and increased BBB permeability in mice [163]. This effect could be relevant, since magnetic resonance imaging studies in patients with posttraumatic epilepsy demonstrated that the site of increased BBB permeability colocalized with the presumed epileptic focus [164] and animal studies found a positive correlation between the extent of BBB opening and the number of seizures [165].

Pharmacological inhibition of LOX using dual inhibitors of LOX/COX pathway phenidone [160, 166], which decreased the production of CysLTs, or BW755C [167] attenuated the seizure activity. Similarly, zileuton was shown to decrease spike-wave discharges in pilocarpine epileptic rats [168], strongly suggesting that leukotrienes play a role in epilepsy.

In line, montelukast and 1,2,3,4, tetrahydroisoquinoline, a LTD4 synthetic pathway inhibitor, suppressed the development of kindled seizures, as well as pilocarpine-induced spontaneous recurrent seizures in mice [162]. Bay-u9973, a nonselective CysLT receptor antagonist, montelukast, and pranlukast increased the latency to generalized seizures and decreased the mean amplitude of electroencephalogram (EEG) recordings during seizures in PTZ-injected mice [163]. Furthermore, montelukast prevented the PTZ-induced BBB disruption and leukocyte infiltration.

Clinical evidence highlights the efficacy of pranlukast in patients with intractable partial epilepsy. In fact, pranlukast reduced seizure frequencies probably normalizing MMP-9 in serum, reducing leakage of proinflammatory cytokines into CNS, and inhibiting extravasation of leucocytes from brain capillaries [22].

## 3. Conclusion

The interest in the field of LT research was traditionally focused on their effects on asthma and allergic disorders. Over the years, accumulating data have highlighted the involvement of these inflammatory mediators—and in particular of the CysLTs and their receptors—in a broader range of inflammation-associated diseases. Among them, the presence of elevated levels of CysLTs in CNS lesions, the evidence that polymorphisms within the LT biosynthesis pathways are associated with an increased risk of cerebral pathological events and the accumulating data obtained in animal studies, also suggested a role for CysLTs in cerebrovascular diseases.

Robust data sustain the role of this pathway in brain ischemia; nevertheless, to elucidate the involvement of the CysLT pathway in the other neurodegenerative disorders, further efforts, in experimental and clinical investigation, are needed. The antileukotriene drugs had been approved for the treatment of asthma more than 20 years ago, and promising evidence indicate their beneficial effects in the treatment of neurodegenerative disease. They show a limited toxicity and a good therapeutic-to-toxic ratio; nevertheless, before hypothesizing a translation to clinic, further studies are needed to underlie their molecular mechanism(s) and demonstrate the potential clinical benefits in the treatment of CNS disease. Moreover, remains to explore how other receptors able to bind the CysLTs, such as GPR17, could influence the development of CNS disease and to define their eventual therapeutic value.

## Figures and Tables

**Table 1:** The neuroprotective effects of drugs acting on CysLT pathway in CNS disorders.

Brain ischemia				
Model	Drug class	Molecule	Effect	Reference
Transient MCAO in gerbils	5-LOX inhibitor	AA-861	↓ neuronal death	[70, 71]
Transient MCAO in rats	5-LOX inhibitor	Minocycline	↓ ischemic injuries, IgG exudation, and neutrophils and macrophage/microglia accumulation	[83]
Permanent MCAO in rats	FLAP inhibitor	MK-886	↓ acute infarct size	[72]
Permanent MCAO in rats	5-LOX inhibitor	Zileuton	↓ edema, infarct volume, neurological deficits, MPO activity, lipid peroxidation levels, inflammatory reaction, and apoptosis	[73–75]
OGD in rats astrocytes	FLAP inhibitor	MK-886	↓ astrocyte proliferation and death	[29]
OGD in rats astrocytes	5-LOX inhibitor	Zileuton	↓ astrocyte proliferation and death	[29]
OGD in rats astrocytes	5-LOX inhibitor	Caffeic acid	↓ astrocyte proliferation and death	[29]
Transient MCAO in rats and mice	CysLTR-1 antagonist	Pranlukast	↓ neurological deficits, infarct volume, BBB disruption, neuron loss in the ischemic core, astrocyte proliferation in the boundary zone, and ischemia-induced glial scar formation ↑ motor-sensory recovery	[15, 65, 68, 78]
Permanent MCAO in rats and mice	CysLTR-1 antagonist	Pranlukast	↓ neurological deficits, infarct volume, edema, BBB disruption, neuron degeneration, and MPO-positive neutrophil accumulation	[49]
Transient MCAO in rats and mice	CysLTR-1 antagonist	Montelukast	↓ infarct size, brain atrophy, neuron loss, behavioural dysfunction, oxidative stress, inflammation, release of glutamate, apoptosis, and lactate dehydrogenase activity	[80, 81]
Permanent MCAO in rats and mice	CysLTR-1 antagonist	Montelukast	↓ infarct volume, brain edema, neuron density, and neurological deficits	[6, 79]
Neonatal hypoxic-ischemic brain damage	CysLTR-1 antagonist	Montelukast	↓ ischemic cerebral and nerve damage ↑ behavior recovery of chronic ischemic brain damage	[82]
OGD in rats astrocytes	CysLTR-1 antagonist	Montelukast	↓ astrocyte proliferation	[29]
Transient MCAO in rats	CysLTR-2 antagonist	HAMI 3379	↓ neurological deficits, lesion volume, edema, and neuronal degeneration and loss	[50, 69]
OGD in PC12 cell	CysLTR-1/CysLTR-2 dual antagonist	Bay-u9773	↓ apoptosis	[62]
OGD in rats astrocytes	CysLTR-2 antagonist	Bay CysLT2	↓ astrocyte death	[29]
OGD in rats astrocytes	CysLTR-1/CysLTR-2 dual antagonist	Bay-u9773	↓ astrocyte proliferation and death	[29]
Alzheimer's disease				
Model	Drug class	Molecule	Effect	Reference
Tg2576 mice	FLAP inhibitor	MK-591	↓ A*β* peptide (A*β*) deposition, *γ*-secretase complex, neuroinflammation, and microglia and astrocytes activation	[120]
N2A-APPswe cells	FLAP inhibitor	MK-591	↓ A*β* peptide (A*β*) deposition, *γ*-secretase complex	[120]
Tg2576 mice	5-LOX inhibitor	Zileuton	↓ A*β* peptide (A*β*) deposition, *γ*-secretase complex	[121]
N2A-APPswe cells	5-LOX inhibitor	Zileuton	↓ A*β* peptide (A*β*) deposition, *γ*-secretase complex	[121]
3xTg mice	FLAP inhibitor	MK-591	↓ A*β* peptide (A*β*) deposition, behavioural deficits, neuroinflammation, and microglia and astrocytes activation	[127]
Tg2576 mice	FLAP inhibitor	MK-591	↓ brain tau phosphorylation	[128]
Rat hippocampal neurons treated with A*β*_1–42_	5-LOX inhibitors	NDGA, AA-861	Prevention of neuronal injury and accumulation of ROS	[129]
Microinfusion of A*β*_1–42_	CysLTR-1 antagonist	Montelukast	Improvement of memory impairment via inhibiting neuroinflammation and apoptosis	[125]
Mouse cortical neurons treated with A*β*_1–42_	CysLTR-1 antagonist	Pranlukast	Reverse A*β*_1–42_-induced cognitive deficit and AD features	[130]
Microinfusion of A*β*_1–42_	CysLTR-1 antagonist	Pranlukast	↓ apoptosis	[130]
Mouse neurons treated with A*β*_1–42_	CysLTR-1 antagonist	Montelukast	↓ proinflammatory factors and the apoptosis-related proteins	[131]
Microinfusion of A*β*_1–42_	CysLTR-1 antagonist	Pranlukast	Improvement of memory impairment via inhibiting neuroinflammation and apoptosis	[132]
Parkinson's disease				
Model	Drug class	Molecule	Effect	Reference
MPTP-treated mice	FLAP inhibitor	MK-866	↓ toxicity of dopaminergic neurons; ↑ [^3^H]-dopamine up-take	[137]
MPP^+^ treated SH-SY5Y cell line	FLAP inhibitor	MK-866	↓ toxicity of dopaminergic neurons ↑ [^3^H]-dopamine uptake and cell survival	[137]
LPS-treated mice	5-LOX/COX inhibitor	Phenidone	↓ oxidative stress, microglial activation, and demise of the nigral dopaminergic neurons	[139]
LPS-treated mice	5-LOX inhibitor	Caffeic acid	↓ dopaminergic neurodegeneration and microglia activation	[139]
Multiple sclerosis/experimental autoimmune encephalomyelitis				
Model	Drug class	Molecule	Effect	Reference
PLP-induced EAE mice	5-LOX inhibitor	Zileuton	Delay of the onset and reduction of cumulative EAE severity	[152]
MOG-induced EAE mice	5-LOX inhibitor	Zileuton	Delay of the onset and reduction of cumulative EAE severity	[153]
Cuprizone-treated mice	FLAP inhibitor	MK-886	↓ axonal damage, motor deficits, and neuroinflammation	[149]
MOG-induced EAE mice	CysLTR-1 antagonist	Zafirlukast	↓ CNS infiltration of inflammatory cells and symptoms of EAE	[148]
MOG-induced EAE mice	CysLTR-1 antagonist	Montelukast	↓ demyelination, leukocyte infiltration, secretion of IL-17, permeability of the BBB, chemotaxis of T cells, and severity of EAE	[148]
MOG-induced EAE mice	Dual inhibitor of LOX/COX pathway	Flavocoxid	↓ CNS infiltration of inflammatory cells, infiltration and differentiation of Th1+ and Th17+ cells, and symptoms of EAE	[154]
Epilepsy				
Model	Drug class	Molecule	Effect	Reference
Kainic acid rat model	5-LOX/COX inhibitor	Phenidone	↓ seizure activity, neurotoxic signs, neuronal loss, lipid peroxidation, and protein oxidation	[160, 166]
Kainic acid rat model	5-LOX/COX inhibitor	BW755C	↓ severity of seizures and neurotoxicity	[167]
Pilocarpine rat model	5-LOX inhibitor	Zileuton	↓ spike–wave discharges	[168]
PTZ-mice model	CysLTR-1 antagonist	Montelukast	↓ recurrent seizures, frequency of daily seizures, BBB disruption, leukocyte migration, and mean amplitude of EEG recordings during seizures. ↑ increased the latency to generalized seizures	[162, 163]
PTZ-mice model	*γ*-Glutamyl transpeptidase inhibitor	1,2,3,4, Tetrahydroisoquinoline	↓ kindled seizures and frequency of daily seizures	[162]
Pilocarpine mice model	CysLTR-1 antagonist	Montelukast	↓ kindled seizures and frequency of daily seizures	[162]
Pilocarpine mice model	*γ*-Glutamyl transpeptidase inhibitor	1,2,3,4, Tetrahydroisoquinoline	↓ recurrent seizures and frequency of daily seizures	[162]
Electrically kindled seizure mice	CysLTR-1 antagonist	Montelukast	↓ recurrent seizures and frequency of daily seizures	[162]
Electrically kindled seizure mice	*γ*-Glutamyl transpeptidase inhibitor	1,2,3,4, Tetrahydroisoquinoline	↓ recurrent seizures and frequency of daily seizures	[162]
PTZ-mice model	CysLTR-1 antagonist	Pranlukast	↓ seizure susceptibility and mean amplitude of ictal EEG recordings	[163]
PTZ-mice model	CysLTR-1/CysLTR-2 dual antagonist	Bay- u9773	↑ increased the latency to generalized seizures ↓ mean amplitude of EEG recordings during seizures	[163]
Patients with intractable partial seizures	CysLTR-1 antagonist	Pranlukast	↓ seizure frequencies, leakage of proinflammatory cytokines into CNS, and extravasation of leucocytes, normalizing serum MMP-9	[22]
